# Correlates of current cigarette smoking among school-going adolescents in Punjab, India: results from the Global Youth Tobacco Survey 2003

**DOI:** 10.1186/1472-698X-8-1

**Published:** 2008-01-14

**Authors:** Seter Siziya, Adamson S Muula, Emmanuel Rudatsikira

**Affiliations:** 1 Department of Community Medicine, University of Zambia, School of Medicine, Lusaka, Zambia; 2 Department of Community Health, University of Malawi, College of Medicine, Blantyre, Malawi; 3 Departments of Epidemiology and Biostatistics and Global Health, Loma Linda University, School of Public Health, Loma Linda, California, USA

## Abstract

**Background:**

Smoking is a leading cause of morbidity and mortality globally. There is therefore need to identify relevant factors associated with smoking among adolescents in order to better tailor public health interventions aimed at preventing smoking.

**Methods:**

We used data from the Global Youth Tobacco Survey (GYTS) conducted in 2003 in Punjab, India, on 2014 adolescents of whom 58.9% were males. We conducted a weighted logistic regression analysis, adjusting for age and sex, to determine associations between predictor variables and current tobacco smoking status.

**Results:**

A total of 2014 adolescents participated in the survey in 2003, and of these 58.9% were males. Male respondents tended to be older than females (21.2% of males, and 13.1% of females were of age 16 years or above). The percent of males and females in the other age groups were: 23.0% and 28.6% for <14 years, 27.3% and 31.0% for 14 years, and 28.4% and 27.0% for 15 years, respectively. The following factors were positively associated with smoking: adolescents who received pocket money; adolescents who had parents who smoked, chewed or applied tobacco; adolescents who said that boys or girls who smoke or chew tobacco have more friends; adolescents who said that smoking or chewing tobacco makes boys look less attractive; adolescents who said that there is no difference in weight between smokers and non-smokers; adolescents who said that smoking makes one gain weight; and adolescents who had most or all of their closest friends who smoked. The factors that were negatively associated with smoking were: adolescents who said that boys or girls who smoke or chew tobacco have less number of friends; adolescents who said that girls who smoke or chew tobacco are less attractive; and adolescents who had some of their closest friends who smoked.

**Conclusion:**

The observed associations between current smoking on one hand and peer smoking, and perception that boys who smoke are less attractive on the other, deserve further studies. The factors reported in the current study should be considered in the design of public health interventions aimed to reduce adolescent cigarette smoking.

## Background

Tobacco smoking among adolescents is of public concern because of the immediate and long-term health sequelae such as asthma, chronic cough, cancers, chronic obstructive airways disease and cardiovascular diseases [1-3]. In the past decade data on adolescent smoking behaviours have been accumulating, partly due to work done by the Global Youth Tobacco Survey (GYTS) collaborative group and its partners [4-7]. The prevalence of smoking cigarettes among school going adolescents in India using the GYTS data of 2000 and 2001 have been reported to vary from one State/Union to another between 0.5% in Gao and 22.8% in Mizoram, with the North-Eastern States/Unions having higher rates than the South-Western States/Unions [4]. Current cigarette smoking was defined as having ever smoked even one puff in the past 30 days preceding the study. Jindal et al [8] have reported that the prevalence of having ever smoked in Northern India was lowest in Punjab (2.9% for boys and 1.5% for girls) and highest in Chandigarh (8.5% for boys and 9.8% for girls).

Knowledge of the prevalence of smoking among adolescent is important in estimating the burden of the problem and facilitates evaluation of public health interventions as change in prevalence over time can be assessed. However, prevalence alone without information on the predictors of smoking will fail to provide further indicators that may be useful in targeted-interventions given the scarcity of public health resources. It is therefore important also to identify socio-demographic factors that are associated with cigarette smoking.

There have been reports elsewhere that gender, having parents or friends who were smokers, body image consideration and feeling of acceptance among peers may be important factors associated with smoking among adolescents [9-12]. We therefore designed the current study to assess whether these factors were also associated with current smoking among adolescents in the 2003 Punjabi GYTS.

## Methods

The Punjabi GYTS conducted in 2003 was a cross sectional study, that was aimed to recruit school-going adolescents of ages 13 to 15 years using a two-stage probability sampling technique. In the first stage, primary sampling units were schools which were selected with a probability proportional to their enrolment size. In the second step, a systematic sample of classes in the selected schools was obtained. All students in the selected classes were eligible to participate. An 85-item questionnaire was used and included 'core GYTS' and other additional questions as has been described elsewhere regarding the GYTS methodology [4-6,13].

Data analysis was performed using a Statistical Package for Social Sciences (SSPSS) 14.0 for windows (Chicago, Illinois, United States). For the estimation of prevalence or proportions, the GYTS data are weighted to adjust for design effect (selection of school and class levels), non-response (school, class and student levels), and post-stratification of the sample population relative to the grade and sex distribution in the total population. A similar method of analysis has been used previously [4] and is reported by the CDC in the GYTS Dataset Help File [14]. Weights were applied to effectively resize the sample so that it is representative of the population from which it is sampled from. The weighting factor is given by the formula:

W = W1 * W2 * f1 * f2 *f3 *f4

where W1 = the inverse of the probability of selecting a school

W2 = the inverse of the probability of selecting a classroom within a school

f1 = a school-level non response adjustment factor calculated by school size category (small, medium, large)

f2 = a class-level non response adjustment factor calculated for each school

f3 = a student-level non response adjustment factor calculated by class

f4 = a post-stratification adjustment factor calculated by sex and grade

For the purposes of this assessment we aimed to assess whether parental or friends' smoking status, perceptions of body image and acceptance among peers were associated with current smoking status. We also assessed the association between adolescent perception that individuals who smoke have more friends and their smoking status.

## Results

### Characteristics of the study participants

A total of 2014 adolescents participated in the survey in 2003, and of these 58.9% were males. Male respondents tended to be older than females (21.2% of males, and 13.1% of females were of age 16 years or above) The percent of males and females in the other age groups were: 23.0% and 28.6% for <14 years, 27.3% and 31.0% for 14 years, and 28.4% and 27.0% for 15 years, respectively. About 0.4% of the respondents reported that both parents or guardians used tobacco in any form (e.g. chew, smoke), and 13.6% reported that only the father used tobacco, while only one adolescent reported that only the mother used tobacco.

### Factors associated with smoking

Overall, 3.3% of all respondents were current cigarette smokers. Boys were 1.18 (95%CI 1.16, 1.20) times more likely to be smokers than girls. Compared to adolescents of age <14 years, those of ages 14, 15 and 16+ years were 30% less likely (OR = 0.70, 95%CI 0.68, 0.71), 25% less likely (OR = 0.75, 95%CI 0.73, 0.77), and 55% more likely (OR = 1.55, 95%CI 1.52, 1.59), respectively, to be smokers.

Table 1 shows factors that were associated with current cigarette smoking. The following factors were positively associated with smoking: Adolescents who received pocket money were more likely to be smokers compared to adolescents who did not receive pocket money (OR = 1.25; 95%CI 1.23, 1.26). Adolescents who had parents who smoked, chewed or applied tobacco were more likely to be smokers compared to adolescents who did not have parents who smoked or chewed tobacco (OR = 1.34; 95%CI 1.32, 1.37). Respondents who said that boys or girls who smoke or chew tobacco have more friends were more likely to be smokers compared to those who said that boys or girls who smoke or chew tobacco are not different from non-smokers in the number of friends they have (OR = 2.74; 95%CI 2.67, 2.82, and OR = 1.34; 95%CI 1.31, 1.38, respectively). Adolescents who said that there is no difference in weight whether one smokes or not, and those who said that smokers gain weight were 1.19 (95%CI 1.16, 1.23) and 1.47 (95%CI 1.44, 1.51), respectively, times more likely to be smokers compared with those who said that smoking makes one lose weight. Adolescents who said that boys who smoke or chew tobacco are less attractive were 15% (OR = 1.15; 95%CI 1.13, 1.17) more likely to smoke cigarettes compared to those who said that there was no difference, or they were more attractive than non-smokers. Lastly, adolescents who had most or all of their closest friends who smoked were 3.83 (95%CI 3.72, 3.95) times more likely to smoke compared with adolescents who had none of their closest friends who smoked cigarettes.

**Table 1:** Factors associated with current smoking status among school going adolescents in Punjab, India.

**Factor**	*Adjusted OR (95% confidence intervals)
**Received pocket money in a usual month**	
No	1
Yes	1.25 (1.23–1.26)
**Parents use tobacco**	
No	1
Yes	1.35 (1.32–1.37)
**Felt boys who used tobacco had more friends**	
No difference between smokers and non-smokers	1
Have fewer friends	0.84 (0.81–0.86)
Have more friends	2.74 (2.67–2.82)
**Felt girls who used tobacco had more friends**	
No difference between smokers and non-smokers	1
Have fewer friends	0.43 (0.42–0.44)
Have more friends	1.34 (1.31–1.38)
**Perception on attractiveness of boys who smoked**	
No difference between smokers and non-smokers	1
Less attractive	1.15 (1.13–1.17)
**Perception on attractiveness of girls who smoked**	
No difference between smokers and non-smokers	1
Less attractive	0.87 (0.86–0.89)
**Perception that smoking makes one lose or gain weight**	
Lose weight	1
No difference between smokers and non-smokers	1.19 (1.16–1.22)
Gain weight	1.47 (1.44–1.51)
**Closest friends smoke**	
None of them smokes	1
Some of them	0.73 (0.72–0.75)
Most or all of them smoke	3.83 (3.72–3.95

*weighted analysis adjusted for age and sex

The following factors were negatively associated with smoking cigarettes: Adolescents who said that boys or girls who smoke or chew tobacco have less number of friends were 16% (OR = 0.84, 95%CI 0.81, 0.86) and 57% (OR = 0.43; 95%CI 0.42, 0.44), respectively, less likely to be smokers when compared with those that said that there was no difference in the number of friends one had between smokers and non-smokers. We also found that adolescents who said that girls who smoke or chew tobacco are less attractive were 13% (OR = 0.87; 95%CI 0.86, 0.89) less likely to be smokers compared to those who said that there was no difference in attractiveness between smokers and non-smokers, or smokers were more attractive than non-smokers. Finally, adolescents who had some of their closest friends who smoked were 27% (OR = 0.73, 95%CI 0.72, 0.75) less likely to be smokers compared to adolescents who had none of their closest friends who smoked.

## Discussion

Overall the prevalence of smoking was 3.3%, with more males than females being smokers. Higher prevalence of smoking among males compared to females have been reported in some settings [15], but not demonstrated in other settings [16]. This suggests that the socio-cultural factors that impact on smoking may be different from one setting to the other.

The finding that receiving pocket money was associated with smoking in the current study has also been reported by Siziya et al [17] in Kafue, Zambia, and by Mohan et al [18] in Kerala, India. As we have argued before [17], having disposable cash may influence adolescents to spend the money on buying cigarettes.

In the current study, adolescents who perceived that girls who smoke are less attractive were less likely to be smokers compared to those who thought smoking made no difference, or made an individual look more attractive. This result contradicts that reported by Croghan et al [19], who conducted a study among college students in the United States and found that perceived lower body image satisfaction and low self-esteem were associated with smoking. However, our other finding that adolescents who felt that boys who smoke are less attractive were more likely to smoke compared to those who felt that there was no difference between boys who smoked and non-smokers, or those who felt that boys who smoked were more attractive, supports the finding of Croghan et al [19]. The observed contradictions in the results may be a reflection of how body image is perceived differently between males and females in different societies.

We also assessed whether adolescents' perception of body weight was associated with current smoking. The hypothesis was that adolescents who thought that smoking would make one lose weight were more likely to be smokers as has been demonstrated in Western populations and Japan [20-23]. We, however, found that adolescents who believed that smoking makes one gain weight were more likely to be smokers. This finding is different from what has been reported in Western countries where adolescent smokers generally believe smoking makes one lose weight and hence more attractive. There could be a difference in the definition of attractiveness in Punjab which is different from the predominant Western belief. In Malawi for instance, Bentley et al [24] have reported that study participants indicated a large body silhouette as desirable compared to a smaller build. This is a setting where human immunodeficiency virus (HIV) and malnutrition are leading health problems and thinness is associated with disease and or poverty.

Compared with respondents who said that there is no difference between smokers and non-smokers in terms of the number of friends they have, respondents who felt that boys or girls who smoke have more friends were more likely to smoke, and those who said that boys or girls who smoke have less number of friends were less likely to smoke cigarettes. Although we obtained these consistency results, we are unable to suggest any causality between current smoking and perception of having more friends if one was a smoker. This observed association deserves further study.

Our study also found that adolescents who had parents, and most or all of their closest friends who were smokers were more likely to be smokers themselves. This finding has also been reported elsewhere [17,25,26]. Although parental smoking may influence adolescents to start smoking, we cannot conclude the same for peer smoking. It may be that adolescents who smoke choose other adolescents who smoke to be their closest friends [27].

## Limitations of the study

Our study had several limitations. Our study was based on self report and therefore subject to respondent recall and deliberate misreporting. The study also recruited only school-going adolescents who may not have been representative of the out of school adolescents. The findings of the study may also be limited by not controlling for unmeasured confounders and effect measure modifiers [28]. For example, Bergen et al [29] have reported that perceived academic performance was associated with tobacco use among adolescents. We did not account for perceived academic performance. Finally, data on current smoking were only collected through interviews and not verified by biomarkers such as cotinine assessment or exhaled carbon monoxide [30-33]. However, the study used validated standard GYTS methodology, and weighted analysis that adjusted for design effects and non-responses, to ensure valid comparisons of our findings to other studies using the same methodology. Brener et al [34] has also reported that adolescents in the United States reported reliably on health risk behaviours. The extent to which the reliability obtained by Brener et al, can be extrapolated to the Punjab adolescent group is not known.

## Conclusion

The observed associations between current smoking on one hand and peer smoking, and perception that boys who smoke are less attractive on the other deserve further studies. The rest of the factors reported in the current study should be considered in the design of public health interventions aimed to reduce adolescent cigarette smoking.

## List of Abbreviations used

GYTS: Global Youth Tobacco Survey

HIV: Human immune-deficiency virus

SPSS: Statistical Package for Social Sciences

## Competing interests

The author(s) declare that they have no competing interests.

## Authors' contributions

SS conducted data analysis and participated in the interpretation of the findings and drafting of the manuscript.

ASM sourced the data, interpreted the findings and led the drafting of the manuscript.

ER participated in data analysis, interpretation of the findings and drafting of the manuscript.

All authors have read and approved the final version of the manuscript.

## Pre-publication history

The pre-publication history for this paper can be accessed here:


